# How to improve eRehabilitation programs in stroke care? A focus group study to identify requirements of end-users

**DOI:** 10.1186/s12911-019-0871-3

**Published:** 2019-07-26

**Authors:** Manon Wentink, L. van Bodegom-Vos, B. Brouns, H. Arwert, S. Houdijk, P. Kewalbansing, L. Boyce, T. Vliet Vlieland, A. de Kloet, J. Meesters

**Affiliations:** 1 Department of Orthopaedics, Rehabilitation Medicine and Physical Therapy, Leiden University Medical Centre, Leiden, The Netherlands; 2 Department of Medical Decision Making, Leiden University Medical Centre, Leiden, The Netherlands; 3 Basalt, The Hague, The Netherlands; 4 Faculty of Health, Nutrition and Sports, The Hague University for Applied Sciences, The Hague, The Netherlands; 5 Faculty of Health, Amsterdam University for Applied Sciences, Amsterdam, The Netherlands; 6 Basalt, Leiden, The Netherlands

**Keywords:** Stroke, Telerehabilitation, Rehabilitation, Patients, Caregivers, Perspective, Requirements, Preferences, Co-design

## Abstract

**Background:**

A user-centered design approach for eHealth interventions improves their effectiveness in stroke rehabilitation. Nevertheless, insight into requirements of end-users (patients/informal caregivers and/or health professionals) for eRehabilitation is lacking. The aim of this study was to identify end-user requirements for a comprehensive eHealth program in stroke rehabilitation.

**Methods:**

Eight focus groups were conducted to identify user requirements; six with patients/informal caregivers and two with health professionals involved in stroke rehabilitation (rehabilitation physicians, physiotherapists, occupational therapists, psychologists, team coordinators, speech therapist). The focus groups were audiotaped and transcribed in full. Direct content analysis was used to identify the end-user requirements for stroke eHealth interventions concerning three categories: accessibility, usability and content.

**Results:**

In total, 45 requirements for the accessibility, usability and content of a stroke eRehabilitation program emerged from the focus groups. Most requirements concerned content (27 requirements), followed by usability (12 requirements) and accessibility (6 requirements). Patients/informal caregivers and health professionals each identified 37 requirements, respectively, with 29 of them overlapping.

**Conclusions:**

Requirements between stroke patients/informal caregivers and health professionals differed on several aspects. Therefore, involving the perspectives of all end users in the design process of stroke eRehabilitation programs is needed to achieve a user-centered design.

**Trial registration:**

The study was approved by the Medical Ethical Review Board of the Leiden University Medical Center [P15.281].

## Introduction

Stroke, or a cerebrovascular accident (CVA), often occurs when a clot in the blood vessel blocks the blood flow to the brain cells (ischemic stroke) or when a blood vessel in the brain breaks or ruptures (hemorrhagic stroke). Subsequently, brain cells are deprived of oxygen and glucose, causing damage to the brain tissues. Although stroke mortality rates have decreased in Western countries, the prevalence of stroke is increasing [1].

Stroke survivors can experience lasting impairments with disruption of psychological and social well-being, including activities of daily life, cognitive and emotional functioning and social relationships [2, 3]. Therefore, stroke rehabilitation is a comprehensive, multi-dimensional process including multiple interventions aimed at individual treatment goals in impairment, activity or participation [4], which involves both the patient, their informal caregivers and various health professionals (e.g. physicians, physical and occupational therapists, speech-language pathologists, psychologists) [5].

EHealth is proposed as a useful tool to improve efficiency and quality of rehabilitation care [6, 7]. Ehealth is defined as ‘the use of Information and Communication Technology (ICT) to improve or support interventions in health care’. Consequently, (the effectiveness of) use of eHealth in rehabilitation, also known as eRehabilitation, after stroke has become a research area of interest [8–12]. Examples of stroke eRehabilitation programs used in studies are serious brain games, virtual reality or telerehabilitation [8, 13, 14].

Despite widespread agreement about the potentials of eRehabilitation, eHealth interventions often do not match with the requirements of intended users (e.g. patients and health care professionals), impairing their adoption in health care [15]. Therefore, a ‘user-centered design approach’, in which the requirements/needs of end users are taken into account at each stage of the design of a new product, intervention or service is highly recommended [16–22]. Requirements of end users involved in stroke care (patients, their informal caregivers and different health professionals) can be identified by means of qualitative research (e.g. interviews, focus groups, brainstorm sessions) [23, 24].

Studies assessing requirements/needs of intended users of an eRehabilitation program [17, 25–32] found concerning the content, that interventions should be adapted to the patients’ own circumstances [25, 30, 31] (including personal goals [17, 27]), should deliver rewarding feedback [32] and need to demonstrate outcomes on training performances [25, 27, 28, 31]. Moreover, eRehabilitation programs must be user-friendly [17, 25, 28, 31] (e.g. size of buttons, colors, information delivery, instructions etc.).

However, requirements should not only be identified for the content and usability of eRehabilitation programs, but also for their accessibility in order to enable successful adoption of eRehabilitation in health care [33, 34]. This is important since easy access of eHealth technology (accessibility) allows users to start using it and the extent to which the technology can be used allows users to achieve specified goals (usability) so that users benefit from the services provided (content). Moreover, it is argued that identifying user requirements for eHealth should go beyond functional and technical requirements and also needs to consider requirements for accessibility and acceptability [34–37].

In addition, the requirements in these studies mainly addressed only one aspect of stroke recovery (e.g. hand function, upper limb rehabilitation, weight shifting) or one technology tool (e.g. a game, robotica) [25, 27–31] and not to a comprehensive eRehabilitation program in which multiple modalities are delivered. Although there have been some studies focusing on this area [38–40], requirements of intended users for comprehensive eRehabilitation that covers multiple aspects of stroke management are rather unknown.

Therefore, the aim of this study was to identify the requirements of end users (patients, their informal caregivers and health professionals) for the content, usability and accessibility of a comprehensive eRehabilitation program in stroke care.

## Methods

### Design

To identify the requirements for eRehabilitation in stroke care, a qualitative focus group study was employed with end users. In this study end users were patients with stroke, their informal caregivers and health professionals involved in stroke care. Focus groups are a useful method to gather information about perceptions of participants and to identify perceived requirements of subgroups [41]. The study took place between January 2016 and March 2016 in two rehabilitation centers in the Netherlands, both providing inpatient and outpatient multidisciplinary stroke care: Rijnlands Rehabilitation Center (RRC) in the city of Leiden and Sophia Rehabilitation (SR) in the area of The Hague.

## Recruitment and inclusion

### Patients and informal caregivers

**Fig. 1:**
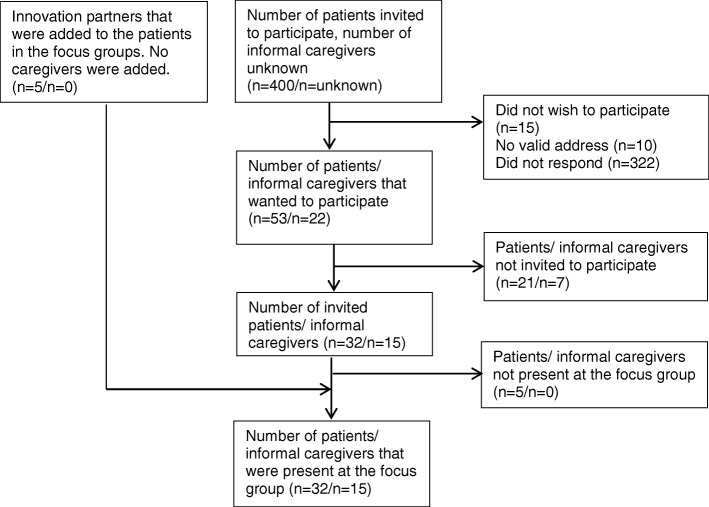
Flow of inclusion of participants in the focus group study

Patients were recruited based on the following criteria: > 18 years, diagnosed with stroke, completed rehabilitation which started after June 2011. From a group of circa 2.700 potential participants which are treated in one of the two rehabilitation centers, 200 patients of each rehabilitation center were randomly selected (Fig. 1). Those 400 patients received a letter with information about the study and an invitation to participate from their former rehabilitation physician. Invitations to patients were directed to the informal caregiver as well, which could be a partner, child, parent or friend who is involved in the daily life of the patient. In addition, a group of five former stroke patients from SR (innovation partners), who come together on a regular basis to discuss the newest innovations in rehabilitation, were invited.

The invitation included a self-developed questionnaire concerning marital status (single, married, divorced, widow/widower), daily activities ((un) paid job, household tasks, student), education level (low: up to and including lower technical and vocational training, medium: up to and including secondary technical and vocational training, and high: up to and including higher technical and vocational training and university), impairments as a consequence of stroke (physical, communication, cognition), use of ICT-devices (smartphone, tablet, laptop, pc) and the purpose of this use (applications, email, information, games, exercises). From the patients/informal caregivers that indicated their willingness to participate, we purposively selected both man and women, young (< 20 age) and older (> 70 age) patients, patients with less (e.g. using a computer only for mail) and more experience with digital devices (e.g. using a smartphone/tablet for applications) and patients with communication, cognitive and physical impairments. Patients with aphasia or severe cognitive problems were asked to bring their informal caregiver in order to help them represent their perspective.

### Health professionals

Health professionals from the two rehabilitation centers (*n* = 56, 29 at Sophia Rehabilitation, 27 at Rijnlands Rehabilitation Centre) were invited to participate by e-mail and selected based on the following criteria: a practiced and certified health professional (rehabilitation physician, physical therapist, occupational therapist, speech therapist and/or a psychologist) with ≥ 2 years of working experience in multidisciplinary stroke care or working as a coordinator of a multidisciplinary team. A selection was made based on availability and profession, so that each profession was represented.

## Data collection

### Focus groups

It was planned to execute four focus groups at the two Rehabilitation centers, two with patients/informal caregivers and two with health professionals. Separate groups were organized with patients/informal caregivers vs health professionals in order to allow patients/informal caregivers to speak freely about experiences in the rehabilitation center and professionals to share their opinions about delivery of care. The aimed group size was 6 to 8 participants, although higher invitation rates were used to account for participants who would decline last minute [42, 43].

A moderator (MW; Msc, female), assistant (BB; Msc, female/HB; Msc, female) and observer (SH; physiotherapist, male/PK; MD, female) conducted the focus groups. The assistant contributed with questions, made sure all participants were involved in the discussion and managed the tape-recorders and time. The observer observed and took notes. The moderator and assistant had no involvement in patient care and a master’s degree in Health Sciences/Human Movement Sciences including education about conduct of interviews. The moderator was trained in communication skills (listening, summarizing and disquisition). Patients/informal caregivers received travel costs reimbursement and were rewarded for participating with a gift card of 10 euro.

### Interview guide

A semi-structured interview guide was developed with open-ended questions concerning three categories of eRehabilitation: accessibility, usability and content. These categories were based on research findings. Existing eHealth frameworks as a theory and guidance for the focus groups were considered by the research team*,* for instance the ‘Technology Acceptance Model’ [44], the ‘Comprehensive Health Technology Assessment Framework’ [45] and the ‘Evaluation of e-health services: user’s perspective criteria’ [46]. However, most frameworks focus on the evaluation process [37, 45–49] instead of the development process of eHealth technologies [50–53], and although the frameworks for development of eHealth admitted user requirements should be identified in an early stage, none of these frameworks described which aspects to explore.

In relation to the focus in this study, accessibility was defined by the research team as “easy access to eRehabilitation for all end users, including patients with disabilities as a consequence of stroke”. Usability was defined as “the extent to which the eRehabilitation service can be used by the specified users (patients, informal caregivers and health professionals) to achieve specified goals with effectiveness, efficiency and satisfaction (e.g. recovery after stroke) in a specific context of use (e.g. during their stay in the rehabilitation center and/or at home)” [35]. Content was defined as “everything end users want to include in eRehabilitation (e.g. services, information, applications, etc.) to achieve specified goals for eRehabilitation in their rehabilitation process.”

Examples of questions included were: “what ICT devices would you like to use for eRehabilitation?” (accessibility), “what aspects would make eRehabilitation easy to use?” (usability) and “what elements of care should be included in an eRehabilitation program?” (content). Prompts were included in the interview guide (e.g. example of eRehabilitation, pictures, etc.) to facilitate participants in verbalizing thoughts.

The interview guide was tested in a pilot focus group with a group of former stroke patients. No adjustments were made to the interview guide and therefore data from this focus group were included in the analysis. The focus groups lasted 2 h, including breaks, and were audiotaped and transcribed in full.

### Participants

Out of the 400 patients and their informal caregivers (200 at RRC/200 at SR) invited to participate in this study, 53 patients (27 at SR/26 at RRC) and 22 informal caregivers (11 at SR/11 at RC) responded. Reasons for non-response were not recorded. Of the 53 responded patients, 32 were invited to participate of whom 27 were present at the focus groups (Fig. 1). Five innovation partners were also present, so that a total of 32 patients participated. Of these patients, 15 had an informal caregiver that participated with them in the focus groups. Seven out of these 15 caregivers were required to support a patient with aphasia or severe cognitive problems. In total 56 health professionals (29 at SR/27 at RCC) were invited, from which 22 responded (11 at SR/11 at RRC). Nine professionals were not able to attend the focus groups, so that eventually 13 professionals participated in the study (7 at SR/6 at RRC).

In total, eight focus groups were conducted; six with patients/informal caregivers and two with health professionals. The characteristics of all patients, informal caregivers and health professionals are presented in Table 1.

**Table 1:** Participants of the focus groups, including the pilot focus group, exploring end-user requirements for eRehabilitation in stroke care

	Patients	Informal caregivers	Professionals
Number of participants	32^a^	15	13
Gender, male; number (%)	19 (59)	4 (27)	3 (23)
Age in years; mean (SD)	57 (15)	61 (10)	–
Time since stroke in months; mean (SD)	28 (14)	–	–
Physical impairment; number (%)	20 (63)	–	–
Problems with communication; number (%)	16 (50)	–	–
Cognitive impairment; number (%)	24 (75)	–	–
Using digital devices (laptop, tablet, smartphone) in daily life; number (%)	32 (100)	–	–
Purpose of using digital devices; number (%)^b^:			
Access to email	18 (56)		
Access to applications	15 (47)		
Searching information	10 (31)		
Playing games	14 (44)		
Doing exercises	8 (25)		
Profession; number (%):			
Physiotherapist	–	–	3 (23)
Psychologist	–	–	1 (8)
Occupational therapist	–	–	3 (23)
Speech therapist	–	–	1 (8)
Rehabilitation physician	–	–	4 (31)
Team coordinator	–	–	1 (8)

^a^Including the five innovation partners

^b^Patients could give more than one answer to each question

## Ethical issues and approval

Informed consent was obtained from all participants. Participants were informed that all their comments were confidential and would be used to improve rehabilitation treatment. It was explicitly mentioned that participation would not affect future treatment in the rehabilitation center. Collected data were reported in such way that persons could not be identified. Only researchers involved in the data analysis had access to the data. The study was approved by the Medical Ethical Review Board of the Leiden University Medical Center [P15.281]. COREQ guidelines were used for adequate reporting of the study [54].

## Data analysis

The audio-tapes of the focus groups were transcribed in full. Directed content analysis was used [55], in which the three predetermined categories of the interview guide (accessibility, usability and content) were used as guidance for analyzing the data. First, two (MW, BB) out of four researchers (BB, MW, SH, PK) independently highlighted text that appeared to reflect a requirement for eRehabilitation (codes). These requirements were directly classified in one of the prescribed categories (accessibility, usability or content). Second, researcher MW examined the data to determine whether subcategories were needed and requirements with comparable content were merged (subcategories). A new category was added if one of the three prescribed categories were not sufficient for identified requirements. It was aimed to stop conducting focus groups when no additional categories of user requirements were found, indicating saturation [41]. In each step of the analysis, discrepancies were compared and discussed in order to reach consensus. When the two researchers (BB, MW) still disagreed, a third researcher (JM), made a final decision. The framework and illustrations were discussed with two other researchers (LB, JM). Transcripts and findings were not returned to participants for comments. The 2 software package Excel 2010 was used to organize codes, subcategories and categories.

## Results

### User requirements

**Fig. 2:**
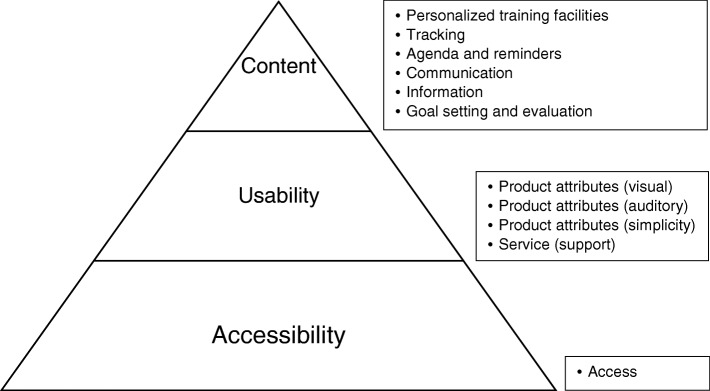
Subcategories of user requirements for the accessibility, usability and content of a stroke eRehabilitation program

In total, 45 user requirements (codes) for a comprehensive eRehabilitation program were identified for the three prescribed categories (accessibility, usability and content). No categories were added, since the three prescribed categories were sufficient for all identified requirements. The requirements were classified into a total number of 11 self-determined subcategories. Most subcategories and requirements were identified for Content (6 subcategories/27 requirements), followed by Usability (4 subcategories/12 requirements) and Accessibility (1 subcategory/6 requirements). The 11 subcategories are presented in Fig. 2. An additional table presents the user requirements for eRehabilitation in stroke care for each (sub) category (Table 2).

**Table 2:** Requirements for an eRehabilitation program in stroke care according to patients, informal caregivers and health professionals

Categories	Subcategories	Requirements	Patients/caregivers	Professionals
Accessibility:	Access:	No internet connection is required to use eHealth interventions (offline use). (1)	X	X
		eHealth interventions are accessible without logging on each time. (2)	X	X
		Applicable to most commonly possessed ICT-devices (laptop, tablet and smartphone). (3)	X	
		Access for health professionals to the electronic patient record to stay informed about training results. (4)	X	
		Applicable on computers at the rehabilitation center and synchronization with programs used for the electronic patient record. (5)		X
		Different eHealth interventions should be brought together in one central dashboard. (6)		X
Usability:	Product attributes (visual):	Use of pictograms, symbols and graphics. (1)	X	X
		Non-flashing and tranquil interface. (2)	X	X
		Adjustable lay-out settings (font style, font size, background and colors). (3)	X	
	Product attributes (auditory):	Ability to listen to written text. (4)	X	X
		Sounds for alert or as feedback. (5)	X	
	Product attributes (simplicity):	Limited amount of open webpages as a consequence of using a service. (6)	X	X
		Limited amount of information on a single screen. (7)	X	
		Limited options on a single screen to click further to another screen. (8)	X	
	Service (support):	Menu with frequently asked questions (FAC). (9)	X	X
		Videos with instructions on how to use eRehabilitation. (10)	X	X
		Helpdesk. (11)	X	X
		Direct assistance at home/ workplace. (12)	X	
Content	Personalized training facilities:	Physical exercises. (1)	X	X
		Exercises for cognitive functioning. (2)	X	X
		Speech exercises. (3)	X	X
	Tracking:	Monitor activities in daily living (i.e. what activities and for how long). (4)	X	X
		A video system to record exercises at home. (5)	X	
		Monitor a patients’ health status (e.g. body weight, heart rate function, etc.). (6)		X
	Agenda and reminders:	Insight in the rehabilitation schedule of a patient. (7)	X	X
		A reminder function for scheduled appointments. (8)	X	X
		Scheduled time to use eRehabilitation (digital training). (9)	X	X
		Scheduling appointments with health professionals on the initiative of patients and their informal caregivers. (10)	X	X
	Communication:	Contact with peers (patients) to share experiences on how to cope with having a stroke. (11)	X	X
		Contact with peers (care givers) to share experiences on how to cope with having a relative with stroke. (12)	X	X
		Communication between patients and their informal caregivers and health professionals from a distance (telecommunication). (13)	X	X
	Information:	General information about stroke. (14)	X	X
		Hyperlinks to reliable and relevant web pages for patients with stroke and their informal caregivers. (15)	X	X
		Information about patient organizations. (16)	X	X
		Information on how to cope with consequences of stroke (psycho-education). (17)	X	X
		Descriptions on how to perform daily activities (strategy training). (18)	X	
		Insight in agreements and information discussed during a consult. (19)		X
		Insight in final reports of a patients’ rehabilitation process. (20)		X
	Goal setting and evaluation:	Setting goals for eRehabilitation. (21)	X	X
		Evaluation of goals for eRehabilitation. (22)	X	X
		Feedback about training results (i.e. insight in what is trained, the number of completed training sessions and training outcomes). (23)	X	X
		Feedback on goals (i.e. when a goal is accomplished). (24)	X	X
		Use of clinical assessments for goal setting and goal evaluation. (25)		X
		Use of valid questionnaires for goal setting and goal evaluation. (26)		X
		Compare training outcomes of a single patient with those of other patients. (27)		X

A total number of 45 requirements were retrieved from the focus groups. Thirty-seven requirements were mentioned by patients/informal caregivers (6 for accessibility, 12 for usability and 19 for content) and 37 by health professionals (6 for accessibility, 9 for usability and 22 for content). Thirty-two requirements were overlapping between patients/informal caregivers and health professionals, 8 requirements were unique for patients/informal caregivers and 8 requirements were only mentioned by health professionals. The results will be further explained in the following sections by a description of the identified user requirements for each category within each category.

### Accessibility

#### Access

Most patients are interested in eRehabilitation, but not all patients want to use it for their recovery, because it is not suitable for them (e.g. lack of computer skills or disabilities impairing use of technology). This was also acknowledged by health professionals. [Professional_5: *“It is not realistic to strive for every patient to use eHealth. You can offer it to the people who are willing to use it and have the required skills.”*].

Easy access is important according to all end users to establish effective eRehabilitation interventions. [Patient_11: *“If getting into the program fails the first time you try, then you will be done with it soon.”*]*.* Requirements for easy access were: *no internet connection is needed* (requirement 1) and *logging on is only required once* (requirement 2).

Furthermore, patients/informal caregivers want eRehabilitation to be *applicable to most possessed ICT devices* (requirement 3) and that *their health professionals have access to their electronic patient record to stay informed about training results* (requirement 4).

For professionals it was important that eRehabilitation is *applicable on computers at the rehabilitation center and synchronizes with programs used for the electronic patient record* (requirement 5). Moreover, they stated that *different eHealth interventions should be brought together in one central dashboard* (requirement 6). [Professional_7: *“I would like to argue that people only have one account and that all facilities are directly available via one digital environment.”*].

### Usability

#### Product attributes (visual)

Visual disabilities related to stroke were mentioned (e.g. neglect) and resulted in a list of requirements regarding visual attributes, i.e. *use of pictograms, symbols and graphics* (requirement 1) and *a non-flashing and tranquil interface* (requirement 2). [Patient_18: *“I prefer a light and calm background. Lots of colors and flashing lights onscreen often cause me a headache.”*]*.*

In addition, patients/informal caregivers mentioned *lay-out settings should be adjustable* (requirement 3), so that this can be adapted to the patients’ own preferences. [Caregiver_4: “*I have seen so many differences between patients with stroke. No one is the same. So, I can imagine type of letters, colors and so on need to be adjustable.”*].

#### Product attributes (auditory)

As a consequence of cognitive and speech disabilities (e.g. aphasia), end users mentioned eRehabilitation interventions would be more suitable for stroke patients when *being able to listen to written text* (e.g. instruction of exercises) (requirement 4). [Caregiver_12: *“It would be very helpful for my father if he can listen to instructions instead of reading it himself.”*].

In addition, *sounds for alerts or as feedback* (requirement 5) was a requirement of patients/informal caregivers. They prefer alerts as a reminder (e.g. alarm for training) or for fun (direct feedback whilst training). However, some patients also mentioned irritation and fatigue as negative side effects of sounds.

#### Product attributes (simplicity)

End users required simple eRehabilitation interventions to increase usability: *limited options on a single screen to click further to another screen* (requirement 6) and a *limited amount of information on a single screen* (requirement 7).

Patients/informal caregivers also required a *limited amount of open webpages as a consequence of using a service* (requirement 8) to prevent patients from getting lost*.* [Caregiver_9: *“If I look at my husband when he uses the computer, I think it should be very simple. So not too much text, pictures, things you can click on... otherwise he has no idea what he is doing”*].

#### Service (support)

Support on how to use eRehabilitation was considered crucial for usability according to all end users. Several requirements were mentioned: *menu with frequently asked questions* (requirement 9)*, videos with instructions* (requirement 10)*, helpdesk* (requirement 11) and *direct support on location* (requirement 12)*.* [Caregiver_4: *“If you’re at home and you’ll get stuck, you want to be able to ask someone directly for help”*].

### Content

#### Personalized training facilities

End users want eRehabilitation to include tailored training facilities for recovery after stroke, i.e. *physical* (requirement 1)*, cognitive* (requirement 2) and *speech exercises* (requirement 3). [Patient_19: *“If I came to know one thing, it is that no person who have had a stroke is the same, so you should be able to compose it in a way that it applies to you.”*]. Moreover, training facilities need to deliver constant personalized feedback (e.g. symbols, sounds, etc.) to prevent boredom with training and increase fun and accordingly stimulate training adherence and rehabilitation outcomes.

#### Tracking

All end users mentioned activity trackers as an eRehabilitation tool to *monitor daily activities* (requirement 4). [Caregiver_11: *“He forgot to count taking a shower, having breakfast, going up and down the stairs... Then this device measured all activities and he became aware that he actually did a lot. That explained why he was so tired.”*].

Patient/informal caregivers also mentioned *a video system to record exercises* (requirement 5) and the ability to send these recordings to their health professional for feedback. A requirement of health professionals was *monitoring of a patients’ health status (*e.g. *body weight, heart rate function,* etc.*)* (requirement 6).

#### Agenda and reminders

All end users preferred a digital agenda which includes: *a patients’ rehabilitation schedule* (requirement 7)*, a reminder function for scheduled appointments* (requirement 8) and *scheduled time to use eRehabilitation (digital training*) (requirement 9). This was found important in case of cognitive impairments and difficulties with time management after stroke.

Furthermore, patients/informal caregivers want to be *able to make appointments with health professionals on their own initiative* (requirement 10). Professionals required a limit in the number of appointments. [Professional_2: *“I would like if patients can schedule an appointment with me, but only within restrictions. I certainly do not want them to schedule appointments with me every week.”*].

#### Communication

End users required digital communication tools (e.g. chat room, video chat, etc.) in order to facilitate *contact with peers to share experiences on how to cope with having a (relative with) stroke* (requirement 11)*.* [Professional_5: *“There must be a digital function that allows patients and caregivers who have come to know each other in the center, to stay in contact if they want to”*]. Moreover, communication tools can provide *communication between health professionals and patients and their informal caregivers from a distance (telecommunication)* (requirement 12).

#### Information

According to all end users provision of information should include: *general information about stroke* (requirement 13)*, hyperlinks to reliable and relevant web pages* (requirement 14)*, information about patient organizations* (requirement 15) and *information on how to cope with consequences of stroke (psycho-education)* (requirement 16)*.*

In addition, patients/informal caregivers required to get *information on how to complete daily activities (strategy training)* (requirement 18). Professionals want patients/informal caregivers to have insight in *agreements and information discussed during a consult* (requirement 19) and *final reports of their rehabilitation* (requirement 20). [Professional_7: *“Patients easily forget what I have discussed with them during a consult. It would be great if they can have access to this information later. To be honest: To me it is quit strange that some information is still not digitally available.”*].

#### Individual goal setting and evaluation

Requirements mentioned by all end users were: *setting individual goals for eRehabilitation* (requirement 21), *evaluation of these goals* (requirement 22) by getting *feedback about training results* (i.e. insight in what is trained, number of completed training sessions and training outcomes) (requirement 23) and receiving *feedback on goals* (e.g. receiving a digital medal when a goal is accomplished) (requirement 24).

In addition, requirements of health professionals were: *use of clinical assessments to set and evaluate goals* (requirement 25), *use of valid questionnaires to set and evaluate goals* (requirement 26) and *comparing training outcomes of a single patient with those of other patients* (requirement 27). However, they find this irrelevant for patients [Professional_1: *“Comparison of scores is especially useful to me, but not for patients. I am thinking of the prognosis and comparison with the average.”*].

## Discussion

The aim of the study was to identify end-user requirements for the accessibility, usability and content of a comprehensive eRehabilitation program for stroke care. In total 45 user requirements were identified, which were grouped into 11 subcategories. Most requirements of end-users concerned the content of eRehabilitation (27 requirements), followed by usability (12 requirements) and then accessibility (6 requirements).

User requirements were quite similar between patients/informal caregivers and health professionals, but also showed differences in perspectives. For instance, professionals required that eHealth programs are able to run on the computer at their workplace, whereas patients and caregivers mainly want to their smartphone or tablet. This implies that eHealth interventions should be designed in such a way that both requirements are met. Other studies incorporating multiple perspectives [32, 56] did not specifically mention differences between end users, impairing comparability of results.

Compared to previous literature, this study identified new requirements for stroke eRehabilitation interventions. For **Accessibility**, it was found that offline eRehabilitation interventions, brought together in one digital dashboard, need to be directly availability after logging on once. To our knowledge, this was the first qualitative study that found requirements concerning accessibility of stroke eRehabilitation programs.

Identified requirements for **Usability** found in the current study that can be added to the literature were: use of icons/symbols, non-flashing and tranquil interface, ability to listen to written text, methods for simplicity (e.g. limited amount of information on a single screen) and support (a helpdesk, video instructions, etc.). Similar to other studies, all patients had different requirements for lay-out [28, 31]. Thus, design solutions should be tailored to a range of users or need to include lay-out options that users can choose from according to their preferences. Identified requirements for **Content** of eRehabilitation that can be added to the literature were: digital agenda, tracking systems, communication tools, provision of information and goal setting and evaluation. This rather broad range of requirements can be explained by the fact that this study aimed to identify user requirements for a comprehensive eRehabilitation program, instead of a single intervention. Similarities with previous studies were found regarding training facilities, i.e. adaptation to patients’ own preferences and capabilities [17, 25, 27, 28, 30–32] and provision of (rewarding) feedback [25, 27, 28].

A limitation of the study is participants with a certain interest in technology and eRehabilitation were probably more likely to respond, causing response bias and reduced generalizability. Therefore, we used purposive sampling based on the purpose of the use of ICT-devices to capture a broad range of perspectives. The group of patients that participated in the study (responders) did not significantly differ in age and gender from the group non-responders. Another limitation is that the chosen study methodology does not allow for comparisons between subgroups of focus group participants (e.g. different technological abilities), since requirements were studied on the level of the group (patients/informal caregivers and health professionals) instead of the individual participant. However, it would be interesting to know if there are differences in requirements of subgroups and this should be studied in the future. In addition, we could not aim for data saturation amongst health professionals. Data saturation was reached after six focus groups with patients/informal caregivers, but due to practical considerations this was not possible for health professionals. Differences in results between patients/informal caregivers and health professionals may have resulted from this imbalance.

In conclusion, user requirements for an eRehabilitation program for stroke care were identified addressing three categories: content, usability and accessibility. Requirements were to some extent different between stroke patients/ informal caregivers and health professionals. Therefore, involving perspectives of all end users in the design process of eHealth is needed to increase their effectiveness in rehabilitation care. The results in the current study can be used in future studies that apply a user-centered design approach to identify requirements for new eHealth interventions for stroke rehabilitation.

## Data Availability

The datasets used and/or analysed during the current study are available from the corresponding author on reasonable request.
